# Prospectus of North Carolina Journal of Medicine and Surgery

**Published:** 1857-02

**Authors:** 


☞ We have received the "Prospectus of the North Carolina Journal of Medicine & Surgery," too late for notice in the present number—it will be attended to in our next.

